# Administrative cases: an effective alternative to lawsuits in assuring access to medicines?

**DOI:** 10.1186/s12889-019-6529-3

**Published:** 2019-02-20

**Authors:** Virginia Oliveira Chagas, Mércia Pandolfo Provin, Rita Goreti Amaral

**Affiliations:** Federal University of Goiás, 388 Residential Breeze from the Woods 37 St. apt 201 Jataí, Goiás, 75800-000 Brazil

**Keywords:** Judicialization of health, Right to health, Judicial decisions, Pharmaceutical services

## Abstract

**Background:**

Although public policy in Brazil supports access to essential medicines, the health system cannot meet all demand. Increasingly, the population has used legal demands to seek access to medicines, an approach that can undermine equitable access by creating policy inconsistencies (e.g., granting access to medicines outside the SUS formulary). In response, the Executive Branch has signed institutional agreements to create an administrative case for submitting requests for medicines directly to the Executive Branch. The objective of this study was to assess the degree to which the administrative cases for requests are in accordance with public policies and guidelines, e.g., if administrative cases results in fewer decisions to purchase outside the SUS formulary.

**Methods:**

This descriptive study used secondary data from lawsuits filed against the Executive Branch from 2003 to 2015 and from administrative cases granted by the Executive Branch from 2010 to 2015 in the capital of a state located in the central-western region of Brazil. The variables included plaintiffs’ sociodemographic characteristics and diseases as well as the characteristics of the medical products sought via the processes.

**Results:**

Comparing the requests submitted through lawsuits and the administrative cases revealed differences in the incomes of plaintiffs and the costs of medicines. Both methods for submission recorded requests for medicines for diseases of endocrine and circulatory systems; the only difference was the prevalence of diseases of the genitourinary system in the lawsuits. A higher proportion of lawsuits sought medicines outside the SUS formulary with therapeutic alternatives, while medicines outside the SUS formulary without an alternative were more commonly requested in administrative cases.

**Conclusion:**

Administrative cases adhere to the public policies and guidelines of the SUS. The administrative cases results in fewer decisions to purchase outside the SUS formulary with alternative, and more decisions to purchase drugs for which there is a formulary alternative. In addition, administrative cases provide greater equity by favoring lower income applicants. However, administrative cases also reveal deficiencies in the State’s implementation of existing pharmaceutical policies. The public pressure for effective implementation of existing policies may help expand access to medicines.

## Background

In Brazil, the integral right to health is an obligation of the State, written in the Federal Constitution, and it depends on the creation and implementation of public health policies [1]. This Constitution also created the Brazilian Universal Health System (Sistema Único de Saúde – SUS), which is based on the principles of universality, integrality, equality and equity [2].

The principle of universality guarantees access to health services and actions to all people, regardless of socioeconomic characteristics, such as gender, race, income or occupation. Integrality guarantees assistance in all levels of care through promotion, protection and recovery of health. Under the principle of equality, all citizens have their right to health guaranteed by the State in an equal way and must therefore acknowledge the multiplicity and social inequality within the population. Equity, in turn, seeks to give more to those in greater need to compensate for inequalities [2].

However, since the 1990s, Brazilian citizens have had to resort to the judicial system in order to have access to medications and other health goods and services. This approach started with the claims of people living with HIV / AIDS, who faced problems gaining access to medicines and medical procedures [1]. The success of people living with HIV / AIDS in using the legal system to access care may have triggered others to also attempt this strategy as a mechanism for guaranteeing a right and broadening of public policies. This phenomenon is called judicialization of access to medicines [3–5].

The flaws in health policies, insufficient funds for the State to meet the growing demands in health care, and the excessive time it takes, not only for regulatory approval, but also for many drugs to be included in the list of those supplied by the SUS, are the attributed causes of this phenomenon. The lawsuits are used by citizens to overcome the inaccessibility of medicines, caused either by the lack of financial resources to acquire them, by their unavailability in public health services or by the high cost of treatment, especially for the treatment of genetic diseases and neoplastic drugs outside the pharmaceutical care policy and represent an important part of the problem [6, 7].

Unlike many countries with universal coverage, Brazil’s SUS also proposed to guarantee access to essential medicines to meet most priority health needs of the Brazilian population [2]. Since then, laws have been established to provide legal foundations for policies that secure access to essential medicines [8–12].

Essential medicines in Brazil are organized into three component lists: basic, strategic and specialized. The basic component includes medicines for the treatment of diseases that are prevalent and that are treated in primary care services. Under the strategic list, citizens have access to medicines to treat endemic diseases. Finally, the specialized list comprises medicines called for in clinical protocols and therapeutic guidelines [12].

The judicialization of access to medicines has become a national problem that needs to be addressed. In several Brazilian states, the judicial system is being used to make policy decisions that are the responsibility of the Executive Branch. The Executive Branch is responsible for developing and executing public drug policies, and the Judiciary has formal legitimacy to enforce the right of access to the medicines. These decisions from lawsuits is granted only to the patient filing the lawsuit, but not to all patients requesting this medicine from then on. Decisions are inconsistent, and often override public policy or ignore the SUS guidelines [13].

One of the main criticisms of the judicialization of access to medicines is the distortions it creates in public policies when a judge orders that medicines outside the SUS formulary or medicines without proven efficacy and safety are supplied to a plaintiff [14–16].

Inconsistency in judicial decisions was the basis for a complaint brought to the Superior Court of Justice, while a separate case, presently being examined by the Federal Supreme Court, may generate rules and guidelines to be followed by the judiciary in judicialization cases [17].

Judicialization has negative consequences that can offset the benefits. The phenomenon raises overall health system expenditures and, in the case of unregistered medicines or medicines being used for new indications, it may distort policies created to service the community, rather than sparking a needed process to reformulate existing policies [15].

With this perspective, legal institutions represented by the Judiciary and the Public Prosecutor’s Office approached the Executive Branch to search for better solutions. These offices sought to identify criteria for resolving conflicts and limiting the involvement of the Judiciary in public policy through the creation of institutional agreements. These agreements presented a new strategy for solving demand for medicines through dialogue, thus reducing the possibility of lawsuits and related economic, social and political distortions [18–22].

The institutional agreements established an administrative case to respond to citizens’ demands. This approach created an administrative case through which citizens request medicines that are not available through SUS. The requests are then evaluated by a commission of pharmacists, who consider both medical criteria, health record and compatibility with public health policies. The administrative case has no cost to open a request, and there are indications that it is more accessible to people with lower incomes [23]. The administrative case was viewed as a way to reduce the number of lawsuits [24, 25].

The Table 1 list the differences / similarities of lawsuits vs. administrative cases.

**Table 1:** List the differences / similarities of lawsuits vs. administrative cases

	Lawsuits	Administrative cases
Avenue of appeal	Citizen appeals to the Judiciary Branch	Citizen appeals to the Executive Branch
Cost to citizens	It has cost to open a request	It has no cost to open a request
Limitations	No requirement of the type of service that issued the prescription (prescribed by a SUS clinician or private system clinician)	Requirement prescriptions prescribed by a SUS clinician
Evaluation process and criteria	The requests are evaluated by a judge, who consider the plaintiff’s personal documents, the medical prescription containing the description of the medicines requested.	The requests are evaluated by a commission of pharmacists, who consider both medical criteria, health record and compatibility with public health policies.

The administrative case begins when a plaintiff presents the following documents at the Municipal Secretary of Health (Secretaria Municipal de Saúde – SMS): personal documents, the medical prescription containing the description of the requested medicines(s) and an indication of the type of service that issued the prescription (prescribed by a SUS clinician or private system clinician), as well as a medical report describing the patient’s illness. Each case is evaluated by a team of pharmacists, who analyze and defer orders according to current drug policies. Granted cases are forwarded to the Pharmacy of Health-related Products and Special Medications (Farmácia de Insumos e Medicamentos Especiais-FIME) for dispensing. The patient denied the requested prescription through the Administrative mechanism still have the ability to file a lawsuit to demand the desired medication.

Lawsuits originate in the Judiciary System at the request of a lawyer, public defender or prosecutor, who usually file the plaintiff’s personal documents, the medical prescription containing the description of the medicines requested and the type of service that provided the prescription (prescribed by a SUS clinician or private system clinician), as well as a medical report describing the patient’s illness. If the Judiciary decides a lawsuit in favor of the plaintiff, the application is sent to the SMS, which then forwards a copy to FIME to be filled.

**Fig. 1:**
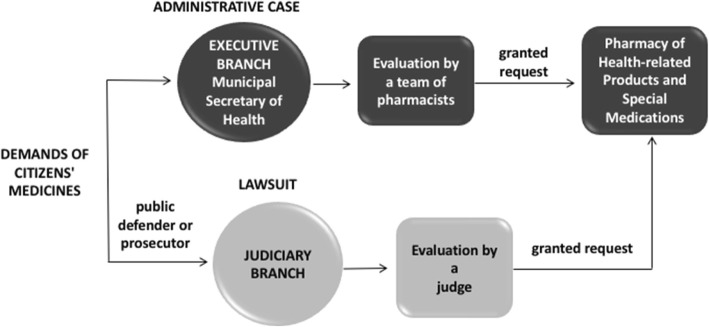
Lawsuits and administratives cases in assuring access to medicines and relations between the involved institutions

In lawsuits, there is no trial with competing experts; instead, the judge decides based solely on the information provided in the plaintiff’s request and the Government has no opportunity to answer the plaintiff’s demand. The government has to comply with the judicial decision within the deadline stipulated by the judge. The lawsuits and administrative cases in assuring access to medicines and relations between the involved institutions is presented in Fig. 1.

The objective of this study was to assess the degree to which the administrative cases for requests are in accordance with public policies and guidelines, e.g., if administrative cases results in fewer decisions to purchase outside the SUS formulary.

## Methods

### Data sources and study setting

This descriptive study was carried out in a state capital located in the central-western region of Brazil where institutional agreements between the Executive Branch and Judiciary were implemented to address the judicialization of access to medicines. Researchers have examined judicialization in this area [5, 26], yet the specific institutional agreements and participation of actors in the justice system has not been well studied.

This study used secondary data on lawsuits requesting medicines from 2003 to 2015 and on administrative cases managed by the Executive Branch from 2010 to 2015. The longer time period for lawsuits was selected to include all lawsuits regarding medicines filed since the first lawsuit, which began in 2003 in the state capital. The five-year period of administrative cases examined was selected to reflect the 2010 signing of an institutional cooperation agreement between the Executive Branch and the Public Prosecutor’s Office. This agreement decreed that all demands for medicines not available under the official SUS lists would be resolved through the creation of an administrative case in the Executive Branch; institutionalizing the administrative case was viewed as a way to reduce the number of lawsuits [24, 25].

The data for the study were collected in July 2016 at a unit within FIME that is responsible for handling lawsuits and administrative cases. Any lawsuits that requested at least one medication were included in the study. Lawsuits or administrative cases with incomplete information or that were granted but never provided the requested medication(s) to the plaintiff were excluded from the analysis.

In total, 3335 lawsuits that requested health goods or services were identified, of which 2557 (76.7%) included a request for at least one medication. Of the 10,631 administrative cases identified, 7192 (67.6%) requested at least one medication.

A simple random sampling procedure was used to select lawsuits for inclusion, stratified by the year of the lawsuit and the administrative case. The random sampling method was applied to both the lawsuits and administrative requests. The sample sizes were determined using a statistical power of 80 (β = 20%), a confidence interval of 95% (α = 0.05) and an accuracy level of 5%.

Through sampling, 568 lawsuits were selected for inclusion in the analysis. Of these, 31(5.4%) were subsequently excluded for incomplete information, and 26 (4.6%) were excluded because, although they were approved, the medication(s) were never delivered to the plaintiff. Ultimately, the sample analyzed contained 511 lawsuits from period under consideration. Of the 495 administrative cases selected, 15 were excluded for incomplete information, and 22 were excluded because the medication(s) were never delivered to the plaintiff, resulting in a sample of 458 administrative cases.

### Variables

The data were collected using a form standardized by the researchers to capture variables investigated in a prior study done in Brazil [22]. Two categories of variables were included: (i) sociodemographic characteristics and the disease(s) of the plaintiffs and (ii) characteristics of the medications solicited via the lawsuit or administrative case.

#### (i) Sociodemographic characteristics and disease(s) of the plaintiffs

The data in this category included age (years); sex (male/female); income (mean monthly income of the head of the household, expressed in U.S. dollars) estimated based on data from the 2010 census on the 63 Territorial Planning Units (Unidade Territorial de Planejamento-UTP) [27]; and disease(s) defined according to the International Statistical Classification of Diseases and Related Health Problems (ICD) [28] as reported in the medical report submitted.

#### (ii) characteristics of the medications solicited through lawsuits and administrative cases

The characteristics of the medicines requested in the lawsuits and administrative cases were the amount of medication(s) requested; the prescription’s origin (were prescribed by a SUS clinician or private system clinician); the classification of the medications according to the Anatomical Therapeutic Chemical Classification (ATC) [29]; and the total cost of the medicines (relative to the sum of the medicines requested based on the price at the Bank of Health Price of the Ministry of Health) [30]. Medications were also classified according to their position with regard to the official lists (it is on the medicines list and is provided free of charge). The categories included [31–36]: (1) within the SUS formulary (belonging to the official lists of medications of SUS); (2) outside the SUS formulary (per the official SUS lists of medications) but with a therapeutic alternative available from SUS through the third level of the ATC classification [29]; or (3) outside the SUS formulary and without a therapeutic alternative available from SUS.

### Statistical analysis

The data were analyzed in the STATA software, version 14.0. The Shapiro-Wilk (SW) test was used to verify the normality of the quantitative variables [37]. A descriptive analysis of the lawsuits and administrative cases in the sample was done, with qualitative variables presented as absolute and relative frequencies and quantitative variables presented as the mean, standard deviation (SD), median and interquartile range (IQR) [38].

## Results

In the 969 cases analyzed, 2315 medicines were requested, including 1501 via lawsuits and 814 in administrative cases.

The Table 2 shows the descriptive and comparative analysis of sociodemographic characteristics and diseases regarding the type of case. There were differences in both the income of the plaintiffs and the average total cost of the drugs when comparing the lawsuits with the administrative cases (*p* <  0.001). Regarding the origins of the prescriptions, a greater proportion of prescriptions in administrative cases were prescribed by a SUS clinician (64%), as opposed to a private clinician (36%).

**Table 2:** Descriptive and comparative analysis of sociodemographic characteristics and diseases regarding the type of case

Variables	Total (*n* = 969)	Lawsuits (*n* = 511)	Administratives cases (*n* = 458)	*p*
Age (years)				
Mean (SD)^1^	44.2 + 24.6	42.8 + 24.7	45.7 + 24.4	0.098^3^
Median (IQR)^2^	50.0 (23.0–64.0)	43.0 (20.0–64.0)	52.0 (26.0–64.3)	
Plaintiffs’ monthly income (US$)				
Mean (SD)^1^	949.18 + 754.47	1058.70 + 827.49	815.32+ 632.78	< 0.001^3^
Median (IQR)^2^	717.97 (498.93–1046.53)	778.81 (551.10–1131.71)	644.95 (438.08–851.83)	
Cost of Medicines(US$)				
Mean (SD)^1^	426.53+ 902.77	353.93+ 1036.91	507.56+ 716.87	< 0.001^3^
Median (IQR)^2^	122.88 (35.03–455.50)	96.92 (34.34–285.99)	205.94 (40.06–670.56)	
Sex				
Male	535 (55.2)	292 (57.1)	243 (53.1)	0.202^4^
Female	434 (44.8)	219 (42.9)	215 (46.9)	
Prescription’s Origin				
Prescribed by a SUS clinician	322 (49.1)	77 (28.2)	245 (64.0)	< 0.001^4^
Prescribed by a private system clinician	334 (50.9)	196 (71.8)	138 (36.0)	
Amount of medication(s)				
Mean (SD)^1^	2.4 + 2.0	2.9 + 2.4	1.8 + 1.0	< 0.001^3^
Median (IQR)^2^	2.0 (1.0–3.0)	2.0 (1.0–4.0)	2.0 (1.0–2.0)	
Diseases^6^				
Certain infectious and parasitic diseases	9 (0.9)	5 (1.0)	4 (0.9)	1.000^5^
Neoplasms	13 (1.3)	7 (1.4)	6 (1.3)	0.936^4^
Diseases of the blood and blood-forming organs and certain disorders involving the immune mechanism	19 (2.0)	4 (0.8)	15 (3.3)	0.005^4^
Endocrine, nutritional and metabolic diseases	235 (24.3)	71 (13.9)	164 (35.8)	< 0.001^4^
Mental and behavioural disorders	82 (8.5)	77 (15.1)	5 (1.1)	< 0.001^4^
Diseases of the nervous system	167 (17.2)	84 (16.4)	83 (18.1)	0.488^4^
Diseases of the eye and adnexa	40 (4.1)	17 (3.3)	23 (5.0)	0.188^4^
Diseases of the ear and mastoid process	4 (0.4)	–	4 (0.9)	0.040^5^
Diseases of the circulatory system	170 (17.5)	109 (21.3)	61 (13.3)	0.001^4^
Diseases of the respiratory system	44 (4.5)	17 (3.3)	27 (5.9)	0.055^4^
Diseases of the digestive system	62 (6.4)	27 (5.3)	35 (7.6)	0.134^4^
Diseases of the skin and subcutaneous tissue	2 (0.2)	–	2 (0.4)	0.223^5^
Diseases of the musculoskeletal system and connective tissue	75 (7.7)	28 (5.5)	47 (10.3)	0.005^4^
Diseases of the genitourinary system	156 (16.1)	139 (27.2)	17 (3.7)	< 0.001^4^
Certain conditions originating in the perinatal period	1 (0.1)	–	1 (0.2)	0.473^5^
Congenital malformations, deformations and chromosomal abnormalities	1 (0.1)	–	1 (0.2)	0.473^5^
Injury, poisoning and certain other consequences of external causes	2 (0.2)	–	2 (0.4)	0.223^5^
External causes of morbidity and mortality	1 (0.1)	–	1 (0.2)	0.473^5^

^1^Standard deviation; ^2^Interquartile range; ^3^*Mann-Whitney* Test; ^4^Pearson’s chi-squared Test; ^5^Fisher’s Exact Test; ^6^Diseases were classified acording to the chapters of the International Statistical Classification of Diseases and Related Health Problems (ICD)

The results of this study demonstrate that the administrative cases are more likely to be filed by lower-income users (*p* <  0.001); predominantly request medicines within the SUS formulary for the digestive and metabolic (*p* <  0.001) and cardiovascular system (*p* <  0.001) (Table 2).

Table 3 shows the descriptive and comparative analysis of the medicines’ characteristics. This analysis suggests that the greatest proportion of demands via either administrative or judicial cases were for endocrine (*p* <  0.001) and circulatory system diseases (*p* = 0.001). The only difference was the number of plaintiffs with genitourinary diseases using judicial cases (*p* <  0.001).

**Table 3:** Descriptive and comparative analysis of medicines characteristics as to the type of case

Variables	Total (*N* = 2315)	Lawsuits (*n* = 1501)	Administratives cases (*n* = 814)	*p*
ATC Classification^1^				
Digestive tract and metabolism	774 (33.4)	416 (27.7)	357 (43.9)	< 0.001^2^
Blood and blood forming organs	134 (5.8)	75 (5.0)	60 (7.4)	0.016^2^
Cardiovascular system	504 (21.8)	414 (27.6)	90 (11.1)	< 0.001^2^
Dermatologicals	23 (1.0)	20 (1.3)	3 (0.4)	0.026^2^
Genito urinary system and sex hormones	59 (2.5)	42 (2.8)	17 (2.1)	0.301^2^
Systemic hormonal preparations	24 (1.0)	15 (1.0)	9 (1.1)	0.809^2^
Antiinfectives for systemic use	31 (1.3)	23 (1.5)	8 (1.0)	0.272^2^
Antineoplastic and immunomodulating agents	17 (0.7)	13 (0.9)	4 (0.5)	0.313^2^
Musculo-skeletal system	107 (4.6)	63 (4.2)	41 (5.0)	0.484^2^
Nervous system	430 (18.6)	314 (20.9)	119 (14.6)	< 0.001^2^
Antiparasitic products, insecticides and repellents	11 (0.5)	11 (0.7)	- (−)	0.010^2^
Respiratory system	82 (3.5)	42 (2.8)	40 (4.9)	0.009^**2**^
Sensory organs	96 (4.1)	47 (3.1)	49 (6.0)	0.001^2^
Various	24 (1.0)	5 (0.4)	18 (2.2)	< 0.001^2^
Classification of the Medicines				
Within the SUS formulary	1032 (44.6)	685 (45.4)	347 (42.6)	0.164^2^
Outside the SUS formulary with alternative	589 (25.4)	430 (28.8)	159 (19.5)	< 0.001^2^
Outside the SUS formulary without alternative	695 (30.0)	386 (25.8)	308 (37.8)	< 0.001^2^

^1^*Anatomical Therapeutic Chemical*; ^2^Pearson’s chi-squared Test

Regarding the classification of medications according to the three categories, lawsuits included a higher proportion of medicines outside the SUS formulary with an SUS therapeutic alternative (*p* <  0.001), while, in administrative cases, there was a higher frequency of medicines outside the SUS formulary without an SUS therapeutic alternative (*p* <  0.001) (Table 3).

## Discussion

Demands for medicines within the SUS formulary submitted via the administrative case show that pharmaceutical policies are not being implemented effectively, as these medicines should be available to the population through SUS. The Committee approves a request for a drug that is covered within the SUS formulary. If the drug should have been available, then there has clearly been a failure in understanding the formulary, or in the inventory management, distribution and purchasing systems of SUS.

Some studies confirm the hypothesis that both the administrative cases and lawsuits have emerged in response to fragmented and ineffective functioning of the health system, such as, the management failures, the shortages of medicines in the health units, the deficiency in the number and quality of medical care in relation to demand, the inability to choose the professional and the healthcare unit providing care, and precariousness in the transfer of information to the citizens, which prolonged the therapeutic itinerary of the users, forcing them to seek the lawsuits. These barriers have already been identified in other studies conducted around the world, including in countries with universal health coverage, revealing the difficulty of overcoming these challenges [3–5, 39].

The administrative case adhered to public policies by granting requests for medicines within the SUS formulary. This approach helps to preserve the principles of universality and equality since it allows all plaintiffs who requested these medicines to receive the standard treatments for the disease. It creates a policy exception, but it may be for a valid reason.

This study’s results indicate that the administrative case is effective in guaranteeing people’s right to access to medicine since medicines provided via this route were mostly either medicines within the SUS formulary or medicines outside the SUS, that are not covered in public formularies. The discrepancy between lawsuits and administrative requests for drugs for the genitourinary system may indicate this may be requests for drugs for erectile dysfunction that are not covered in public and private formularies.

In comparison with lawsuits, administrative cases showed lower demand for medicines outside the SUS formulary with a therapeutic alternative and an increase in the requests for medicines outside the SUS formulary without a therapeutic alternative. These results indicate that the pharmacists responsible for assessing the administrative requests approved more medicines outside the SUS formulary without alternatives. Thus, their decisions adhere to the aim of these cases to address gaps in health care. However, filling gaps in care can deepen social inequities by shifting limited public resources to provide care to a small portion of the population [40].

SUS’ guarantee of the integrality of health care does not imply that the State should provide everything including the medicines outside the SUS formulary [41]. Thus, demands for medicines outside the SUS formulary require a technical analysis to determine whether there are therapeutic alternatives that could be used. The judge does not have the technical knowledge to analyze if a medicine outside the SUS formulary has some therapeutic alternative available in the SUS lists. Thus, it is necessary to evaluate the demands of medicines by a team of pharmacists who have technical knowledge in the area of drug policies [42].

Furthermore, some therapeutic alternatives available through the SUS system may in fact be outdated or of limited benefit to all. There is also evidence of the need to update the official SUS lists, in order to reduce the number of requests for medicines outside SUS formulary [42].

The study found that plaintiffs in lawsuits were higher income than those in administrative cases. Lawsuits do involve initial costs, indicating that plaintiffs used the justice system when they had the resources to pay these expenses [14, 26, 43–45]. It is not uncommon for a plaintiff to request that the judge waive legal fees. Administrative cases, with no initial cost to open a request, are thus more accessible to people with lower incomes [23].

A large part of the Brazilian population faces financial difficulties in acquiring prescription medications [46]. Overall, spending on medicines jeopardizes total health expenditures across different income strata, especially among those with lower incomes, and has an impact on the system’s overall budget. Since there is no reimbursement, it represents a health expense for the health system that has to pay this bill twice; there is no reimbursement from the health plan to the SUS that provided that medicine requested.

Drug spending accounts for between 25 and 35% of the total health expenditures among households in upper income brackets. In contrast, in lower income strata between 60 and 70% of health expenditures are spent on medicines [47].

This analysis suggests that the greatest proportion of demands via either administrative or judicial cases were for endocrine, nervous and circulatory system diseases. The only difference was the number of plaintiffs with genitourinary diseases using judicial cases. These are all chronic diseases, providing additional evidence on the changing health and disease profiles of the Brazilian population and the impact of the changes on the demand for health care [48, 49]. When the care offered through routine access routes is not consistent with the needs of users, it becomes justifiable for plaintiffs to seek out new paths of access [50]. Managers of the Brazilian health system can utilize the data on legal and administrative cases to meet medicinal needs to learn more about the changes in the epidemiological and demographic characteristics of the population and to adapt the system’s emphasis within primary health care [51].

A greater proportion of prescriptions were prescribed by a SUS clinician in administrative cases (64%) compared to judicial cases (36%). This finding is because administrative cases can only be opened with a prescription and medical report issued by SUS providers since 2011. In the case of lawsuits, there are no regulations about the origins of prescriptions. As a result, requests submitted to the justice system are more likely to interfere with the structure and functioning of the health system [52]. Requiring a SUS prescription to open an administrative case results in use of SUS by those who would otherwise seek private sector care. This practice is known as a private public mix, in which plaintiffs seek to use the public system to overcome the lack of coverage of medicines in the private system [53].

The economic implication of the private public mix would be the displacement of the scarce health resources destined to meet the health needs of the population and that depend exclusively on the SUS to fulfill its therapeutic itinerary, to attend people who have private health insurance. Because private health plans do not reimburse the SUS, this practice shifts SUS resources to serve private sector clients [4].

Some requests come directly from SUS patients (more commonly in lower income groups) and some from privately insured patients who go to the SUS for a prescription denied by a private provider or insurer, and only then obtain an SUS prescription (which is presumably constrained by the formulary in many cases).

The results of this study demonstrate that the administrative case reveals a fragile health system with deficiencies in the implementation of policies, such as failures in the supply of health units that should provide medicines within SUS formulary. The requests for medicines within the SUS formulary reveal gaps in operation of public and private sector supply system. Also, requests for medicines within the SUS formulary with therapeutic alternatives may reflect a lack of education of providers/prescribers related to the policy of substitution of medicines. It might also reflect gaps in supply system or funding.

The best approach would be for the government to update and revise drug policies frequently, including or excluding medicines that meet the population’s health needs and not just meet judicial or administrative cases requesting medicines. Both administrative and judicial cases favor individual needs rather than considering the collective needs of the population and overall availability of medicines within the health system. Moreover, these demands promote the shifting of public policy resources to the satisfaction of few, and contributes to unequal access, resulting in a worsening of inequities.

It is up to the public to demand that these deficiencies are remedied rather than continually creating new avenues for access to medicines that only serve a portion of the population.

The actions of pharmaceutical services should encompass all aspects of the system, from the management to the major beneficiary, the patient. It is important that the municipal health manager has a good knowledge of the organizational structure, the demographic and epidemiological profile, as well as the living and health conditions of the local population so that the actions of the pharmaceutical services meet the health needs of the community.

The results of this study are expected to contribute to the revision of existing policies and to the adoption of new measures to improve access to medicines for those who depend on the public health system for treatment.

### Limitations

The limitations of this study were that we didn’t look at the validity or reasoning for why the request for exemption might be approved and another limitation of study is the fact that we only were able to review records in one geographic area.

## Conclusion

It is concluded, therefore, that the administrative cases do adhere to public policies and guidelines of SUS. Compared to judicial cases, administrative cases result in fewer decisions to purchase outside the SUS formulary with alternative, and in more decisions to purchase drugs for which there is a formulary alternative. Administrative cases also provide greater equity by favoring lower income applicants. However, these cases also reveal deficiencies on the part of the State in implementing existing pharmaceutical policies. The public pressure for effective implementation of existing policies may help expand access to medicines.

## Abbreviations

ATC: Anatomical Therapeutic Chemical Classification System
FIME: Pharmacy of Health-related Products and Special Medications
ICD: International Statistical Classification of Diseases and Related Health Problems
IQR: Interquartile Range
SD: Standard Deviation
SUS: Brazillian Universal Health System
UTP: Territorial Planning Units
